# Synthesis and characterization of dual-responsive poly(N-vinylcaprolactam-co-N-methylolacrylamide) nanogels

**DOI:** 10.1080/15685551.2022.2086412

**Published:** 2022-06-09

**Authors:** Noverra M. Nizardo, Dzul Fadli Alimin, Maria L. A. D. Lestari

**Affiliations:** a Department of Chemistry, Faculty of Mathematics and Natural Sciences, Universitas Indonesia, Depok, Indonesia; b Department of Pharmaceutical Science, Faculty of Pharmacy, Universitas Airlangga, Surabaya, Indonesia

**Keywords:** Nanogel, poly(N-vinylcaprolactam), poly(N-methylolacrylamide), lower critical solution temperature, thermoresponsive polymers

## Abstract

This article reports the synthesis of poly(N-vinylcaprolactam-co-N-methylolacrylamide) (P(NVCL-co-NMA)) nanogels and investigates their thermo-/pH-responsive behavior. The formation of nanogels was synthesized using free radical emulsion polymerization by varying the monomer composition of NVCL:NMA, and their molecular structure was characterized by ^1^H-NMR and FTIR. It was found that the nanogels were successfully prepared, and the nanogels exhibited LCST-type phase transition behavior. Cloud point transition temperature (T_c_) was studied as a function of copolymer composition, MBA concentration, and pH of the solution by exploring their changes in turbidity using UV–vis spectrophotometer. Our studies reveal that T_c_ nanogels increased with increasing concentration of NMA, which is due to the hydrophilicity of NMA. Our research also demonstrated that the increase in MBA percentage could decrease the T_c_ of the synthesized nanogels. Interestingly, P(NVCL-co-NMA) nanogels showed not only a thermoresponsive behavior but also a pH response with increasing T_c_ in a strong acidic environment owing to the H-bonds within the polymer chains. The results show that nanogels with initial monomer composition of NVCL and NMA of 75% and 25%, respectively, and using 4% of MBA showed T_c_ around 35°C at pH 7.4. In addition, DLS studies also confirmed this result since the particle sizes became much larger after surpassing the temperature of 35°C. Due to this founding, such nanogels might have potential application in controlled release. Nevertheless, further studies regarding the adjustment of T_c_ are still needed.

## 1. Introduction

Thermoresponsive polymer is one type of stimuli-responsive polymers that has gained interest to be investigated. This type of polymers can respond to the change of temperature. There are two types of thermoresponsive polymers, namely, polymer with lower critical solution temperature (LCST) and upper critical solution temperature (UCST). In the case of LCST-type polymers, the polymer chains are soluble in an aqueous medium at low temperatures but become insoluble while heating, and the transition from coil-to-globule occurred. On the other hand, UCST-type polymers are insoluble at low temperatures, but as the temperature rises, they become soluble [1]. Consequently, UCST-type polymers exhibit a transition from globule-to-coil. The driving force for the phase transition is the polymer–polymer interactions and polymer–water interactions. Thermoresponsive polymers with LCST behavior are the most widely studied so far, with poly(N-isopropylacrylamide) (PNIPAM) as the classical example [2–4]. PNIPAM has cloud point (T_c_) in the range of 32°C in an aqueous medium, which is independent of molar masses [5], and thus, make it an interesting polymer to be explored.

Like PNIPAM, poly(N-vinylcaprolactam) (PNVCL) is a non-ionic polymer that also exhibits T_c_ in the range of 32°C in an aqueous medium, but its T_c_ depends on the molar masses. Moreover, due to better biocompatibility and more stability against hydrolysis compared to PNIPAM, PNVCL becomes a promising candidate as a carrier for drug delivery [6]. Therefore, many studies about the thermoresponsive behavior as well as the possible applications of PNVCL-based polymers in the drug delivery system have been reported [6–8]. Furthermore, different structures of PNVCL-based polymers, i.e., micelles [9], hydrogels [10], microgels [11], as well as nanogels [12,13], have also been investigated as a carrier.

Nanogels are nano-size hydrogels that consist of three-dimensional crosslinked hydrophilic polymers and can swell by maintaining their structure without dissolving [14]. Nanogels have hydrogel characteristics such as the ability to absorb water and also because of their responsiveness to changes of pH and/ or temperature. The addition of a comonomer in the synthesis can be done to adjust the sensitivity of the nanogel to pH and temperature [15]. Moreover, because of their small sizes but high swelling ratio, nanogels have the potential to be used in drug delivery, not only oral route but also topical and via injection [16,17]. Furthermore, by virtue of their nanostructure, as a carrier, nanogels are able to improve the solubility of hydrophobic drugs and deliver their active substances intracellularly [15].

Several studies about PNVCL nanogels have been reported. Macchione et al. synthesized poly(N-vinylcaprolactam) nanogels through free radical polymerization and succeeded in proving that the cloud point transition (T_c_) of PNVCL nanogels decreased with increasing mole percent of MBA as crosslinking agent and NVCL monomer concentration in the reaction mixture [13]. Furthermore, to satisfy the requirement for drug delivery, the T_c_ of such polymers can be adjusted by the addition of a comonomer and control the molar masses. Demirel and Klitzing studied the effect of 2-dimethylaminoethyl methacrylate (DMAEMA) in P(NVCL-co-DMAEMA) nanogels that showed a decrease in particle sizes between 45 and 48°C and exhibited pH-dependent behavior [12]. Özkarahman et al. prepared poly(N-vinylcaprolactam-co-methacrylic acid sodium salt) [p(VCL-co-MMANa)], poly(N-vinylcaprolactam-co-itaconic acid sodium salt), [p(VCL-co-IANa)] copolymeric microgels, and poly(N-vinylcaprolactam-co-methacrylic acid sodium salt-co-itaconic acid sodium salt) [p(VCL-co-MMANa-coIANa)] terpolymeric microgel using precipitation polymerization method and found that hydrophilic co-monomers such as MAANa and IANa might tune the volume phase transition temperature [18].

Another investigation was reported by Sudakhar et al. who successfully synthesized poly(N-vinylcaprolactam-co-hydroxyethylacrylamide) nanogels through free radical emulsion copolymerization and succeeded in proving that the addition of HEMA monomers will increase the cumulative release of curcumin compounds both below and above the nanogel transition temperature [19]. Gonzáles et al. succeeded in synthesizing poly(N-vinylcaprolactam-co-hydroxymethylacrylamide) nanofibers, which were sensitive to temperature, through free radical copolymerization in DMF solvent followed by electrospinning and successfully demonstrated that the LCST of the P(NVCL-co-NMA) copolymer increased with increasing levels of NMA monomer. The obtained copolymer showed temperature-controlled release of rhodamine B [20].

Up to date, the most recent research concerning poly(N-vinylcaprolactam-co-N-methylolacrylamide) (P(NVCL-co-NMA)) was focused on the formation of P(NVCL-co-NMA) hydrogel nanofiber by electrospining by increasing temperature, which was synthesized by free radical solution polymerization [20]. Noteworthy, detailed correlation between monomer ratio and phase transition, in which the investigation performed in relatively acidic, neutral, and basic solution, has not yet been reported. Herein, we synthesized nanogels of P(NVCL-co-NMA) via free radical emulsion polymerization and studied the effect of monomer composition and concentration of crosslinking agent on their phase transition temperature (T_c_) by varying the ratio of NVCL and NMA as well as the concentration of N,N’-methylenebisacrylamide (MBA) as the crosslinker in the monomer feed. Additionally, phase transition temperature in different pH was also investigated. The addition of NMA was designed to adjust the T_c_ of P(NVCL-co-NMA) nanogels in the physiological temperature of 37°C and was expected to be responsible for the pH responsiveness. Moreover, to our knowledge, there is no study that revealed the pH-responsive behavior of P(NVCL-co-NMA) nanogels.

## 2. Experiment

### 2.1. Reagent

N-Vinylcaprolactam (NVCL, 98%) and N,N’-methylenebisacrylamide (MBA, 99%) were purchased from Sigma-Aldrich. Sodium dodecyl sulfate (SDS) was purchased from BASF. Ammonium persulfate (APS) was kindly given by Clariant Indonesia. N-Methylolacrylamide (NMA) was kindly given by Evonik Indonesia. N,N,N*′*,N*′-*Tetramethyl ethylenediamine (TEMED, 99%), dipotassium hydrogen phosphate (K_2_HPO_4_, 98%), and potassium dihydrogen phosphate (KH_2_PO_4_, 99%) were purchased from Merck.

### 2.2. Synthesis of P(NVCL-co-NMA) nanogels

Series of P(NVCL-co-NMA) nanogels were synthesized via free radical emulsion polymerization. In a typical procedure, 1044 mg (7.5 mmol) of NVCL, 253 mg (2.5 mmol) of NMA, 61.7 mg (0.40 mmol) MBA (4% mole of monomer), and 21.5 mg (0.07 mmol) SDS were dissolved in deionized water in a Schlenk flask with stirring. This mixture was then purged using nitrogen for 15 minutes under continuous stirring and followed by the addition of 22.8 g (0.10 mmol) of APS and 11.6 g (0.10 mmol) of TEMED. After that, the mixture was heated at 70°C for 5 hours under continuous stirring. After 5 h, the reaction was stopped by exposing the mixture to the air. The formed nanogels were purified by dialysis and dialyzed against distilled water for 7 days, with the change of water every 24 h in dialysis membrane with a molecular cut-off 12–14 kDa. Afterward, the purified nanogels were dried in an oven at 60°C. This synthesis method was adapted from the work of Macchione et al. [13]. Homopolymer of PNVCL and PNMA were also synthesized using the same procedure. The feed compositions of nanogels are presented in Table 1. The code consists of the monomers, i.e., VC for NVCL and MA for NMA. The number following the monomer code indicates the mol percentage of each monomer in the monomer feed. Meanwhile, the number 4M represents the MBA content in the feed, which is 4% of the total mol percentage of the monomer. For instance, VC75MA25-4M means that there are 75 mol% of NVCL and 25 mol% of NMA with 4% of MBA in the feed.

**Table 1.** Nanogel codes and feed composition and yield ofP(NVCL-co-NMA) nanogels

Nanogel Code	Feed Composition						Yield^a^ (%)
	NVCL	NMA	MBA	APS	TEMED	SDS	
	(mg)	(mg)	(mg)	(mg)	(mg)	(mg)	
PNVCL	1392	0	61.7	22.8	11.6	21.5	44
VC75MA25-4M	1044	253	61.7	22.8	11.6	21.5	57
VC50MA50-4M	696	506	61.7	22.8	11.6	21.5	56
VC25MA75-4M	348	758	61.7	22.8	11.6	21.5	51
PNMA	0	1011	61.7	11.4	5.8	21.5	57
VC75MA25-2M	1044	253	30.8	22.8	11.6	21.5	22
VC75MA25-8M	1044	253	123.3	22.8	11.6	21.5	46

^a^Determined gravimetrically

### 2.3. Measurements and analysis

#### 2.3.1. FTIR

FT-IR spectra of nanogels were obtained using Shimadzu IR Prestige 21. The dried nanogels were finely ground with KBr powder. The measurement was performed between 400 and 4000 cm^−1^ at ambient temperature.

#### 2.3.2. ^*1*^ *H-NMR*

^1^H-NMR spectra were recorded with Bruker-Avance 500 MHz Nuclear Magnetic Resonance instrument using deuterated water (D_2_O) as solvent at 19°C.

#### 2.3.3. Cloud transition temperature

Cloud point transition temperature (T_c_) was measured using Thermo Scientific Multiscan Go UV-vis Spectrophotometer. Solutions of nanogels (3 mg/mL) in varying pH buffers were heated slowly from 15 to 60°C in a water bath, and the transmittance at 600 nm was measured every 5°C. T_c_ of each nanogels is the deflection point of the transmittance versus temperature plot.

#### 2.3.4. Dynamic light scattering

Solutions of nanogels (3 mg/mL) were prepared in phosphate buffer pH 7.4. The Z-Average of nanogels were measured at 5°C interval from 20 to 45°C using Horiba-SZ Particle Size Analyzer SZ-100z (532 nm, 90° scattering angle).

#### 2.3.5. Transmission electron microscopy (TEM)

The transmission electron microscopy sample was prepared by blotting sample on Cu-grid Support Carbon Films. The measurement was carried out using FEI Tecnai G2 20S-Twin operating at 200 keV with a LaB6 filament, and the images were taken using an Eagle™ CCD camera. A sample solution of 3 mg/mL was employed in the measurement. The particle size distribution was plotted as a histogram, and ImageJ 1.53q software was used to measure the particle size diameter.

## 3. Results and discussion

### 3.1. Synthesis and characterization of P(NVCL-co-NMA) nanogels

**Scheme 1.**
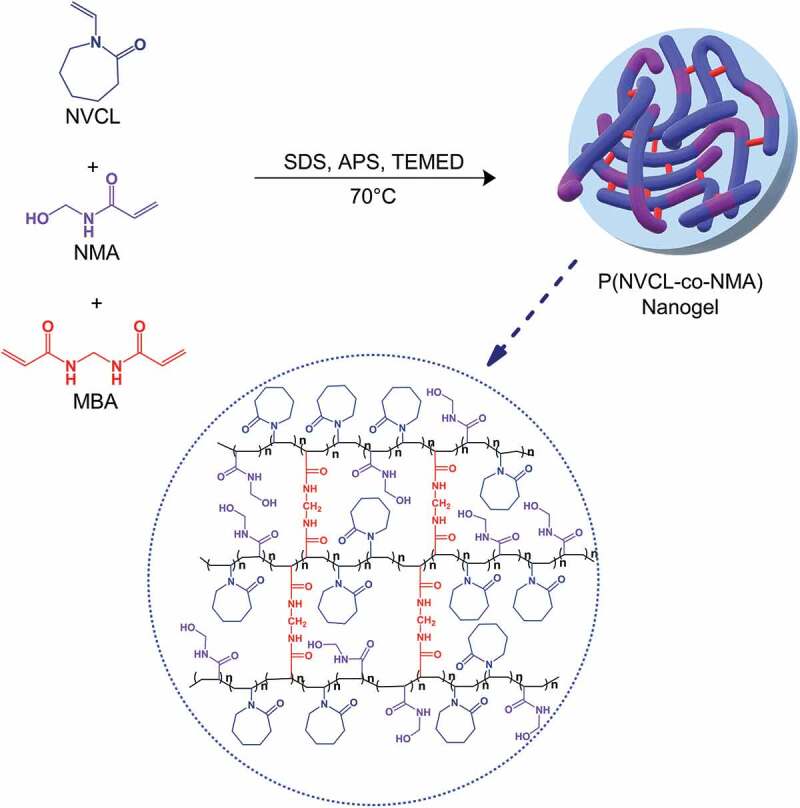
Synthesis of P(NVCL-co-NMA) nanogel.

In this research, a series of nanogels from NVCL and NMA were synthesized via free radical emulsion polymerization using water as solvent and SDS as surfactant (Scheme 1). The polymerization was performed at 70°C, which allowed it to be initialized by thermal initiator APS and TEMED. After purging with N_2_, the reaction mixture appeared to be homogenous. This homogeneity soon changes after reaching polymerization temperature. As the polymer chains grow, their solubility decreased, making the initially homogenous system to be heterogeneous [13]. The nanogels were synthesized by varying the composition of each monomer as well as the initial concentration of MBA as the crosslinking agent. The obtained nanogels were purified via dialysis against distilled water and then dried in an oven to isolate the white solid nanogels.

**Figure 1.**
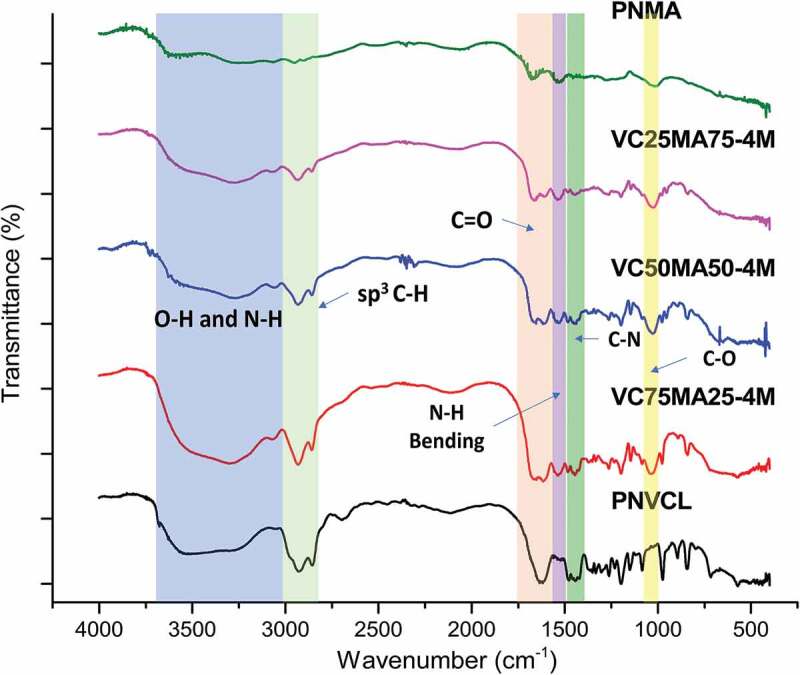
FT-IR spectra of P(NVCL-co-NMA) nanogels with MBA of 4%.

To confirm the success of NVCL and NMA polymerization, nanogels were characterized at their molecular level using FT-IR and ^1^H-NMR. Figure 1 shows FT-IR spectra of P(NVCL-co-NMA) nanogels, which exhibit characteristic bands at 2900–3000 cm^−1^, 1613 cm^−1^, and 1477 cm^−1^ corresponding to stretching vibration of sp^3^ C–H, amide C=O, and C–N, respectively. Weaker transmittance at 1532 cm^−1^ from secondary amide N–H bond is also visible, confirming the existence of MBA crosslink in this nanogel. Other nanogels containing NMA had similar FT-IR spectra with relatively lower intensity at 2900–3000 cm^−1^ with the decrease of initial NVCL composition during synthesis. This could be caused by the smaller amount of sp^3^ C–H bond from NMA pendant. A more intense peak at around 1530 cm^−1^ also appears as an effect of additional secondary amide group in NMA pendant. Nanogels containing NMA also show peaks at around 1046 cm^−1^ corresponding to C–O bond [19].

**Figure 2.**
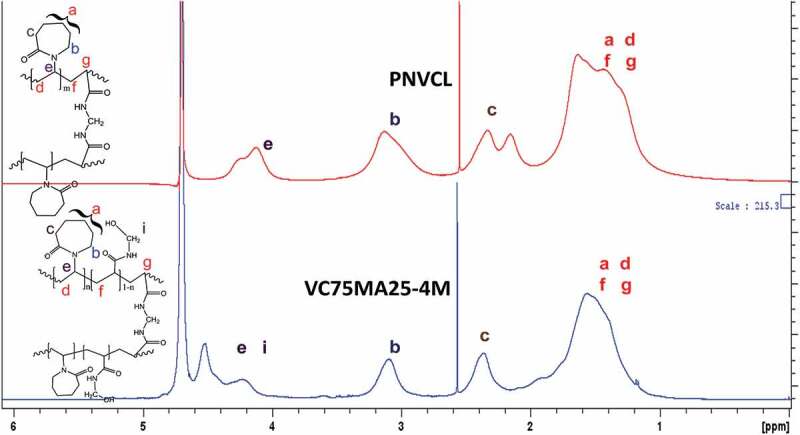
^1^H-NMR spectra of PNVCL and VC75MA25-4M nanogels in D_2_O.

^1^H-NMR spectra of PNVCL and VC75MA25-4M nanogels is displayed in Figure 2. As shown, ^1^H-NMR spectra of PNVCL nanogel exhibited peaks at δ*_H_ *= 1–2 ppm, which corresponds to protons in carbon **a** and polymer backbone of the nanogel. At δ*_H_ *= 2–2.8 ppm, δ*_H_* = 3 ppm, and δ*_H_ *= 4–4.5 each from protons at carbon **c, b**, and **e** respectively. In addition, ^1^H-NMR for VC75MA25-4M exhibited peaks at δ*_H_ *= 1–2.1 for protons at carbon **a** and polymer backbone of the nanogel. At δ*_H_ *= 2.1–2.8 ppm, δ*_H_ *= 2.8–3.2, and δ*_H_ *= 4–4.6 ppm each corresponding to proton at carbon **c, b**, and **e** and **i** in the structure of VC75MA25-4M nanogel. Both PNVCL and VC75MA25-4M showed similiar ^1^H-NMR spectrum to previous studies [13,20]. Moreover, ^1^H-NMR spectrum of VC75MA25-4M enables us to quantify the composition of NVCL and NMA in nanogel, that is 73:27, which is close to the composition added initially during the synthesis (75:25), meaning that the reactivity of NVCL and NMA toward copolymerization is almost equal.

### 3.2. Cloud point transition temperature (T_c_) of P(NVCL-co-NMA) Nanogels

#### 3.2.1. Effects of monomer composition on T_c_ of P(NVCL-co-NMA) nanogels

Thermoresponsive behavior of P(NVCL-co-NMA) nanogels was investigated using UV–vis spectrophotometer with the concentration of 3 mg/mL in phosphate buffer pH 7.4 by heating at 5°C intervals. It is estimated that the nanogels exhibited LCST-type phase transition behavior due to the thermoresponsive properties of PNVCL in aqueous. At low temperatures, the hydrogen bonds occurred between water molecules, and the polymers caused both negative enthalpy of mixing and entropy since the polymer and water are in an ordered state. This makes Gibbs free energy mixing (ΔG_mix_) negative and the polymers soluble in water (or swelling of crosslinked polymers). As the temperature raises beyond the T_c_, the hydrogen bonds break, which leads to the less negative enthalpy. As a consequence, the entropy becomes dominant, resulting in the phase separation that occurred (or shrinking of crosslinked polymers) [21].

**Figure 3.**
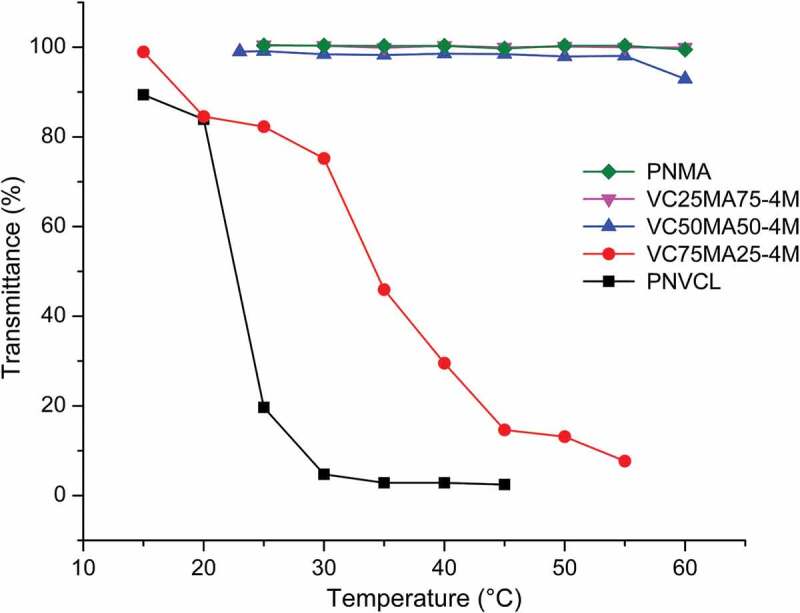
Thermoresponsive behavior of P(NVCL-co-NMA) nanogels with different monomer feed ratio in 3 mg/mL phosphate buffer pH 7.4.

**Figure 4.**
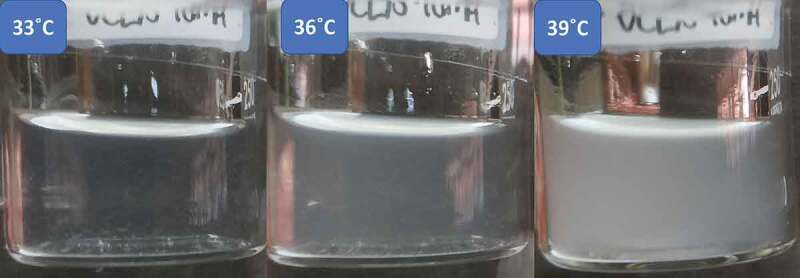
Changes in turbidity of 3 mg/mL VC75MA25-4M nanogel solution near the phase transition temperature.

T_c_ of thermoresponsive polymers can be tuned by adding hydrophilic or hydrophobic co-monomers. Hydrophilic co-monomers shift the T_c_ to higher temperatures because more energy is needed to destroy the hydrogen bonds, while hydrophobic co-monomers showed a contrary effect [22]. Figure 3 shows that all nanogels exhibit LCST-type phase transition behavior, and the T_c_ of nanogels increased with the addition of NMA. PNVCL nanogel exhibited T_c_ at around 25°C and VC75MA25-4M around 35°C (Figures 3, 4). Meanwhile, VC50MA50-4M, VC25MA75-4M, and PNMA nanogel did not show any transition at the given temperature. This fact could be caused by the relatively higher hydrophilicity of the NMA side chain. As a result, with the increase in the amount of NMA, interactions between polymer chains and water molecules are more favorable. This leads to the swelling of nanogels, and thus, releasing water from the nanogels becomes harder as it needs more energy. Hence, a higher value of T_c_ was observed [20,23]. The same trend was also observed in previous studies from Gonzales and Frey, which reported the effect of NVCL and NMA monomer composition in P(NVCL-co-NMA) copolymers on the phase transition temperature of the copolymers. They found that the increasing amount of NMA influenced the rise of T_c_ [20]. Several research groups reported also that the increasing T_c_ could be observed by adding a hydrophilic co-monomer, such as oligo(ethylene glycol) methacrylate or N,N-dimethylacrylamide [24–26]. A similar effect from the hydrophobic pendant point of view on T_c_ of polymer had also been reported by Gao et al. where acrylic acid monomer addition slows down the phase transition of P(NIPAM-co-PAA) copolymers [27].

**Figure 5.**
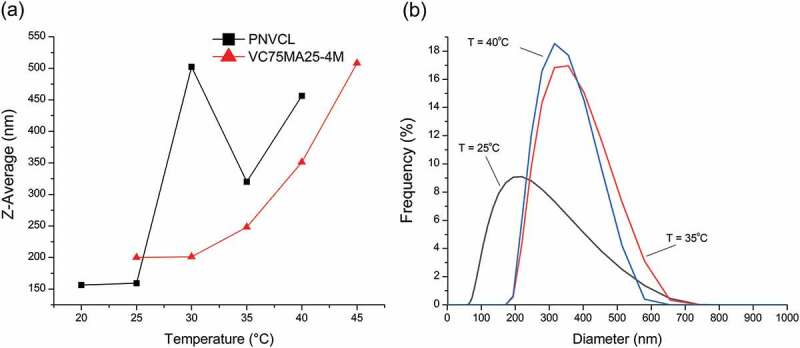
(a) Z-Average particle sizes of PNVCL and VC75MA25-4M nanogels while heating. (b) particle size distribution of VC75MA25-4M nanogel during the phase transition.

**Figure 6.**
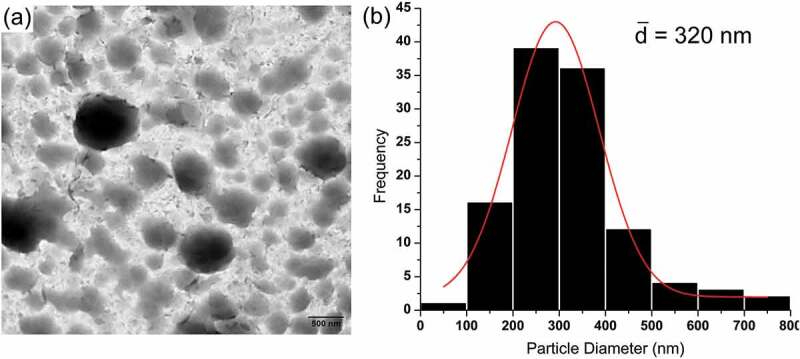
(a) TEM image and (b) particle size distribution histogram of VC75MA25-4 M nanogels.

Figure 5(a) shows the Z-Average particle sizes of PNVCL and VC75MA25-4M nanogels, which are affected by temperature. It was observed that the PNVCL nanogel has a Z-Average of 156 nm at 20°C and experience a significant change in particle sizes after 25°C. In addition, the Z-Average of VC75MA25-4M nanogel at 25°C was 199 nm, and increasing particle sizes were observed started at 35°C. The increase in particle sizes indicates the aggregation of nanogels particles during heating [13,23] and suggests the phase transition occurred. Thus, it can be concluded from the change of particle sizes that the T_c_ of PNVCL and VC75MA25-4M is 25°C and 35°C, respectively. These results are in good agreement with the T_c_ resulting from UV–vis spectrophotometer. Moreover, the TEM image of VC75MA25-4M nanogel confirmed the morphology of nanogel, which appeared to be globular with average particle sizes of about 320 nm with a monomodal particle size distribution (Figure 6).

Furthermore, the particle size distributions of VC75MA25-4M nanogels shifted to the larger diameter with the rise of temperature (Figure 5(b)) and become narrower after surpassing the T_c_. Such phenomenon was also attributed to the LCST-type thermoresponsive behavior of PNVCL that results in a phase transition from hydrated nanogels at 25°C to dehydrated nanogels starting at 35°C. Nanogels began to shrink at 35°C and caused the interaction between the nanogels more pronounced than their interaction with water molecules, which leads to the aggregation of the nanogels [28]. Thus, a larger particle size was observed above 35°C. This result corroborated the thermoresponsive behavior of P(NVCL-co-NMA) nanogels.

#### 3.2.2. Effect of MBA concentrations on T_c_ of P(NVCL-co-NMA) nanogels

**Figure 7.**
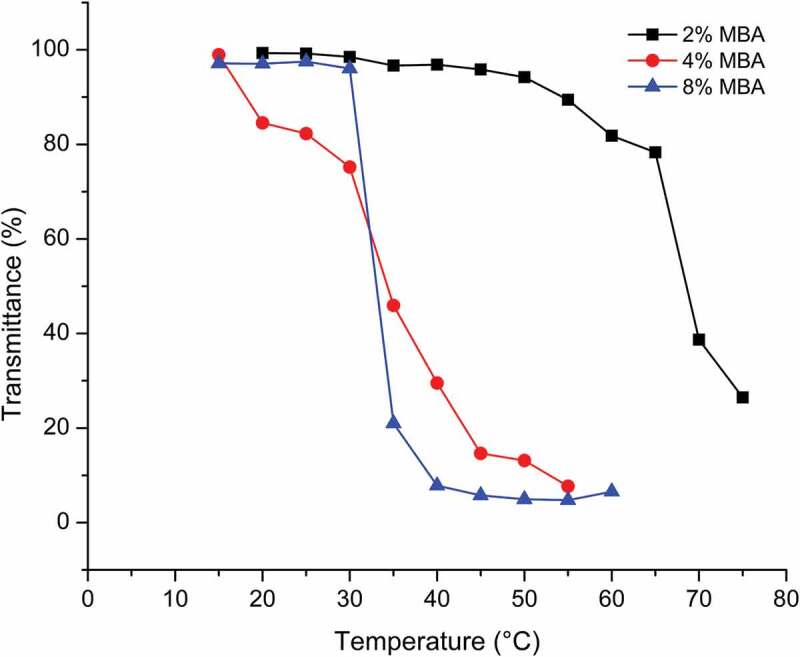
Thermoresponsive behavior of VC75MA25-4M nanogels with different MBA concentration in 3 mg/mL phosphate buffer pH 7.4.

The effect of MBA concentrations on the T_c_ of nanogels is shown in Figure 7. It can be seen that the phase transition temperature of VC75MA25-4M nanogels decreased with increasing MBA concentration. VC75MA25-4M nanogels with 2%, 4%, and 8% of MBA exhibited T_c_ around 65°C, 35°C, and 32°C, respectively, with sharp phase transition shown by 8% of MBA. The same trend was observed in previous studies. In 2021, Romero et al. reported their research, which studied the thermoresponsive properties of P(NVCL-co-PEGDA) nanogels with PEGDA as crosslinking agent and by investigating the effect of PEGDA concentration on T_c_ of the nanogels [29]. As a result, the PNVCL exhibits T_c_ at 32°C, while PNVCL with PEGDA exhibited slight decrease of T_c_ along with increasing PEGDA concentration. This could be reasoned because the increasing crosslinking in the nanogels structure leads to less spaces that can be entered by water molecules. As the distance between polymer chains becomes smaller, intramolecular interactions increase, which gives a further impact on phase separation between the nanogel and water [13,30–32].

#### 3.2.3. Effects of pH on T_c_ of P(NVCL-co-NMA) nanogels

The effects of pH on the nanogel phase transition temperature were also studied by dissolving each P(NVCL-co-NMA) nanogels in a buffered solution of pH 3, pH 7.4, and pH 9 to a concentration of 3 mg/mL. The transmittance for each solution was measured at 600 nm between 15 and 60°C at 5°C intervals. As shown in Table 2, VC50MA50-4M nanogel did not exhibit any phase transition at pH 3. However, it showed a subtle decrease in transmittance starting at 50°C at pH 7.4 and 40°C at pH 9. Interestingly, VC75MA25-4M nanogel showed a transition temperature of around 55°C at pH 3, 35°C at pH 7.4, and 35°C at pH 9.

**Table 2.** T_c_ of P(NVCL-co-NMA) nanogels in 3 mg/mL solution of pH 3, 7.4, and 9

Nanogel Code	Phase Transition Temperature		
	pH = 3	pH = 7.4	pH = 9
	T_c_ (°C)	T_c_ (°C)	T_c_ (°C)
PNVCL	n.d	25	n.d
VC75MA25-4M	55	35	39
VC50MA50-4M	Soluble^a^	55–65^b^	65–75^b^
VC25MA75-4M	Soluble^a^	Soluble^a^	Soluble^a^
PNMA	Soluble^a^	Soluble^a^	Soluble^a^
VC75MA25-2M	n.d	65	n.d
VC75MA25-8M	n.d	32	n.d

^a^Soluble in the entire measurement temperatures

^b^Subtle decrease of the transmittance was observed

**Figure 8.**
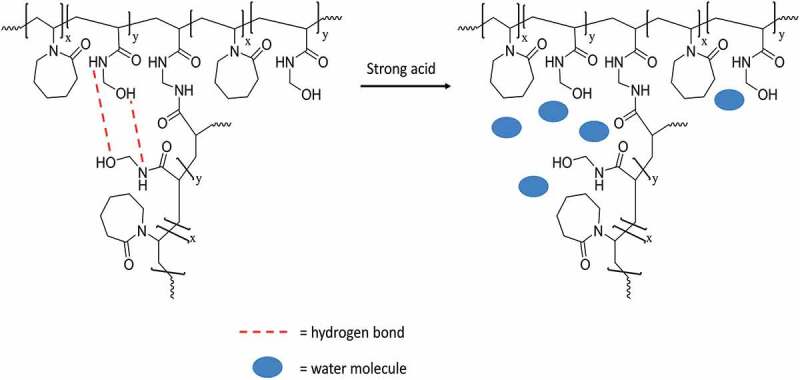
Proposed illustration of pH-responsive behavior of P(NVCL-co-NMA) nanogels in strong acid solution.

Noteworthy, the increase of T_c_ in a strong acidic environment might be attributed to the disappearance of the hydrogen bonds in the amide group of NMA. As illustrated in Figure 8, the amide group in NMA can form hydrogen bonds within the nanogel in the weak acid/ basic medium. The formation of hydrogen bonds, for instance at pH 7.4, prevents the water molecule to penetrate inside the nanogel and makes the nanogel dehydrated more easily, and thus, deswelling of nanogels occurred and decreased the T_c_. Conversely, strong acid might break down the hydrogen bonds within the nanogel, resulting in the hydration of nanogel which makes the nanogels to swell nanoscopic and increased the T_c_ as can be seen at pH 3 [33].

Table 3 shows the effect of pH on the particle size of VC75MA25-4M nanogel. As can be seen, the Z-Average particle size is higher in acidic than in basic environment, which might correlate to the occurrence of protonation of the amide group on the NMA and leads to the increasing ability of the nanogel to swell.

**Table 3.** Z-average particle sizes of VC75MA25-4M nanogels in pH 3 and 9

pH	Z-Average at 40°C (nm)
3	286 ± 19
9	225 ± 36

Phase transition behavior could not be observed within VC25MA75-4M. In fact, PNMA nanogels did not show at any given pH and temperature. Most probably this is due to the higher content of NMA in the nanogels which increases the hydrophilicity of the nanogels. The hydrophilicity may lead to the preferable interaction between the nanogels and the water molecules and make it harder for the nanogels to separate themselves from water molecules and swell [34].

## 4. Conclusion

In this study, a series of P(NVCL-co-NMA) nanogels were synthesized via free radical emulsion polymerization by varying the composition of NVCL and NMA monomers as well as MBA concentration. FT-IR and ^1^H-NMR spectra proved the success of polymerization by showing no absorption peak as well as a chemical shift which belongs to the vinyl group. The greater the NMA amount in the P(NVCL-co-NMA) nanogels is, the higher the T_c_ of the nanogel could be obtained. In addition, the more MBA concentration are added, the lower the T_c_ of the nanogel would be. VC75MA25-4M and VC50MA50-4M nanogels also showed pH-dependent T_c_, in which higher pH would lower the T_c_ of the nanogels.
